# Sirt1 activation by resveratrol is substrate sequence-selective

**DOI:** 10.18632/aging.100542

**Published:** 2013-03-08

**Authors:** Mahadevan Lakshminarasimhan, David Rauh, Mike Schutkowski, Clemens Steegborn

**Affiliations:** ^1^ Department of Biochemistry, University of Bayreuth, Bayreuth, Germany; ^2^ Department of Enzymology, Institute for Biochemistry and Biotechnology, Martin Luther University Halle-Wittenberg, Germany

**Keywords:** resveratrol, Sirt1, Sirtuin, sequence dependent, activation, substrate specific modulation

## Abstract

Sirtuins are protein deacetylases used as therapeutic targets. Pharmacological Sirt1 activation has been questioned since the in vitro activator resveratrol failed to stimulate deacetylation of several physiological substrates. We tested the influence of substrate sequence by analyzing resveratrol effects on Sirt1-dependent deacetylation of 6802 physiological acetylation sites using peptide microarrays. Resveratrol stimulated deacetylation of a small set of sites and inhibited deacetylation of another set, whereas most substrates were hardly affected. Solution assays confirmed these substrate categories, and statistical analysis revealed their sequence features. Our results reveal substrate sequence dependence for Sirt1 modulation and suggest substrates contributing to resveratrol effects.

**One Sentence Summary:**

Testing 6802 acetylation sites reveals that resveratrol effects on Sirt1-dependent deacetylation depend on substrate sequence and suggests substrates relevant for in vivo effects.

Sirtuins are NAD+-dependent protein deacetylases regulating metabolism and aging processes, and they were suggested to mediate lifespan extending effects of caloric restriction [1, 2]. Sirtuin activation through the polyphenol resveratrol can mimic such effects in yeast and higher organisms, and Sirt1 activation can alleviate metabolic diseases in mice [3-5]. Sirtuin activation by resveratrol and other compounds is controversially debated [6, 7], since stimulation of mammalian Sirt1 against the widely used “Fluor-de-Lys” substrate [3] depended on the presence of a non-physiological fluorophor that replaces the peptide chain immediately C-terminal to the acetyllysine [8, 9]. However, a large body of work indicates Sirt1-dependent resveratrol effects [10-12], and peptide sequences terminating with the acetyllysine do not well represent physiological substrate sites.

To test whether physiological deacetylation sites can respond to resveratrol-dependent Sirt1 activation, we employed a mammalian acetylome microarray system. It enables parallel assays on 6802 physiologically occurring acetylation sites, represented by 13-meric peptides with the acetyllysine at the center (Rauh et al., submitted). Sirt1-dependent deacetylation of the microarray peptides was tested in presence and absence of 200 μM resveratrol. Comparing basal deacetylation to the reaction in presence of the compound revealed different outcomes for different substrates (Figure 1A). While deacetylation in presence of resveratrol was significantly increased for some peptides, the majority of sequences showed no or only small changes in deacetylation. Deacetylation of another smaller set of peptides was strongly inhibited.

**Figure 1:**
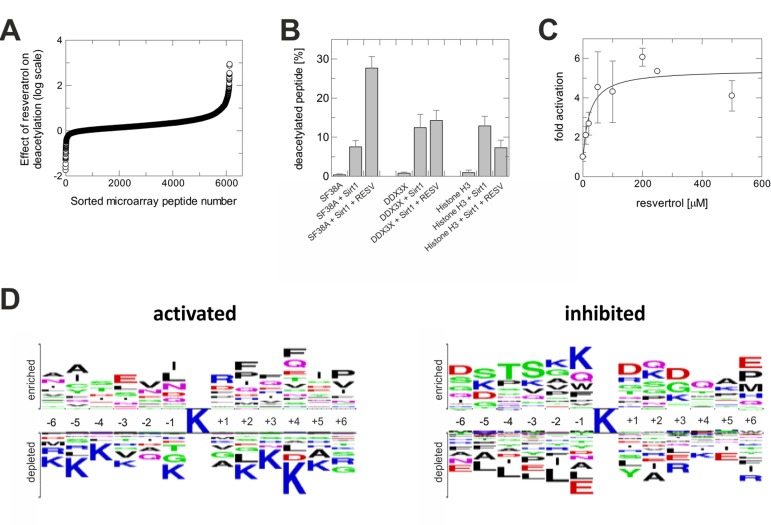
Activation of Sirt1 by resveratrol is substrate sequence-selective (**A**) Sirt1-dependent deacetylation of microarray peptides in presence of resveratrol, relative to their deacetylation without compound. Shown is the negative logarithm of the ratio of residual acetylation signals. (**B**) Effect of resveratrol on Sirt1-dependent deacetylation of peptides from three substrate classes – deacetylation stimulated, unaffected, or inhibited – tested in solution using mass spectrometry. (**C**) Dose-dependent activating effect of resveratrol on the deacetylation of a SF38-K23 peptide by Sirt1. (**D**) Sequence features of peptides whose deacetylation by Sirt1 is either activated or inhibited through resveratrol. The acetyllysine is in the center, residues on top are favored, residues at the bottom disfavored when compared to their overall frequency at this position.

The substrate sequence-dependent effect would explain why resveratrol has Sir2-dependent effects in C. elegans overlapping with, but not identical to, the effects of Sir2 overexpression [13], and why resveratrol failed to stimulate Sirt1 against some substrates [14, 15]. To validate the influence of substrate sequence, we selected peptides representing nuclear deacetylation sites from the groups with increased, unchanged, and inhibited deacetylation in presence of resveratrol. Testing Sirt1-dependent deacetylation of these non-modified, acetylated peptides using mass spectrometry confirmed our conclusion (Figure 1B). Deacetylation of a splicing factor 38A (SF38A)-K23 peptide was activated, deacetylation of a DEAD box protein 3 (DDX3X)-K118 peptide remained unchanged, and deacetylation of a histone H3-K116 peptide was inhibited. Activation of SF38A-K23 deacetylation was stimulated with an EC50 of 22±16 μM (Figure 1C), comparable to the potency of resveratrol against Sirt1 in the FdL assay (EC50~50-100 μM) [3].

The array results indicate Sirt1 substrates whose changed deacetylation might contribute to physiological resveratrol effects: Deacetylation is, e.g., increased for FOXO4-K189 and protein acetyltransferase p300-K489, and decreased for Ku 70-K539 and estrogen receptor alpha-K303. To reveal substrate sequence requirements for resveratrol-dependent activation or inhibition of deacetylation, we statistically analyzed the differently responding substrate groups for amino acid preferences, at each peptide position, compared to the overall occurrence on the microarray (Figure 1D). There is a preference for large, mainly hydrophobic residues at several positions C-terminal to the acetyllysine in substrates whose deacetylation can be stimulated, whereas positively charged residues are disfavored in most positions. For inhibition, hydrophilic residues seem preferential N-terminal and polar and negatively charged residues C-terminal from the acetyllysine.

We identified fluorophor-free, physiological substrate sites whose deacetylation can be stimulated by resveratrol. Our results suggest Sirt1 targets contributing to physiological resveratrol effects and the revealed substrate sequence dependent outcome indicates that Sirt1 inhibition should also be involved. Excitingly, these findings reveal the possibility to develop drugs modulating Sirt1-dependent deacetylation of a single substrate or a small substrate group.

## MATERIALS AND METHODS

### Chemicals

All chemicals were obtained from Sigma (Saint Louis, USA) if not stated differently. Peptides for assays were purchased HPLC purified with unmodified N- and C-terminus from GL Biochem: SF38A-K23, PQYLVE(acetylK)IIRTRI; DDX3X, DRSGFG(acetylK) FERGGN; Histone H3-K116, IHA(acetylK)RVT.

### Cloning, recombinant expression, and purification of Sirt1

Full-length human Sirt1 was cloned into pET15b, resulting in constructs with N-terminal his-tag, and verified by DNA-sequencing. Sirt1 protein was prepared as described [16]. Briefly, protein was expressed in E. coli Rosetta 2(DE3) cells lysed in buffer A (50 mM Tris, pH 7.5, 300 mM NaCl and 10 mM imidazole), and protein bound to Talon resin (Clontech, Mountain View, USA). The resin was washed with buffer A. Protein was eluted with buffer A + 200 mM imidazol, subjected to gel filtration on a Superdex200 column (GE Healthcare) in 25 mM HEPES, pH 7.5, 100 mM KCl, 2 mM DTT, concentrated in amicon units (Millipore), and stored at −80°C.

### Microarray experiments

Acetylome peptide micro-arrays were manufactured as described (Rauh et al., submitted). Shortly, peptides were synthesized on cellulose membranes using parallel SPOT synthesis [17]. Peptides were cleaved from membranes with aqueous triethylamine (2.5 % v/v), solvent evaporated, and peptides re-dissolved in printing solution (70% DMSO, 25% 0.2 M sodium acetate pH 4.5, 5 % glycerol v/v). Peptides were printed on epoxy modified slides (PolyAn), quenched, washed, dried, and stored at 4 °C.

For deacetylation experiments, microarrays were treated 2 h with 20 μg/ml Sirt1 in reaction buffer (25 mM HEPES, pH 7.5, 100 mM KCl, 2 mM dithioerythrol, 3% bovine serum albumin, 1 mM NAD+) in presence or absence of 200 μM resveratrol, washed, and dried. In a hybridization station (HS400, Tecan) at 25 °C, arrays were washed several times, incubated 1 h with three primary antibodies (0.2 μg/ml mouse mAb 7F8 [Abcam], 0.2 μg/ml mouse mAb AcK103 [CST], 0.2 μg/ml rabbit mAb AcK2-100 [CST]) and washed. After incubation with two secondary fluorescence labeled antibodies (0.5 μg/ml Goat IgG Dylight 649 anti mouse and anti rabbit, respectively [Thermo Scientific]) for 30 min, microarrays were washed, dried, and scanned at 10 nm resolution using a Genepix Scanner 4000B (Molecular Devices) and a 635 nm laser. Spot recognition was done using Genepix Pro 7.0 (Molecular Devices). Data processing was done in GNU R (http://www.r-project.org/). Sequence feature logos were created using TwoSampleLogo [18].

### Mass spectrometry deacetylation assays

Assays containing 50 μM NAD+, 5 μM peptide, and 25-200 nM Sirt1 were incubated in 25 mM HEPES, pH 7.5 at 37 °C, aliquots stopped after different time points by adding formic acid, and substrate and product quantified by liquid chromatography coupled mass spectrometry [19]. For stimulation, 1 % (v/v) DMSO (control samples) or 100 μM resveratrol was added. Resveratrol titration was done analogous with DMSO kept at 1 % (v/v), but with 500 μM NAD+ at 25 °C. Deacetylation speed or product levels after 30 min were determined from linear fits to time series, and data shown are representatives of at least three repetitions.

## SUPPLEMENTARY MATERIALS


